# Supplementary material for: Intergenic RNA mainly derives from nascent transcripts of known genes
Source: Genome Biol. 2021 May 5;22:136. doi: 10.1186/s13059-021-02350-x (PMC8097831; doi:10.1186/s13059-021-02350-x)
Supplement: Supplementary file 1 — Additional file 1. Integrated supplementary Figures and Tables. Contains figures from S1 to S5 and tables from S1 to S4. [file 13059_2021_2350_MOESM1_ESM.pdf]

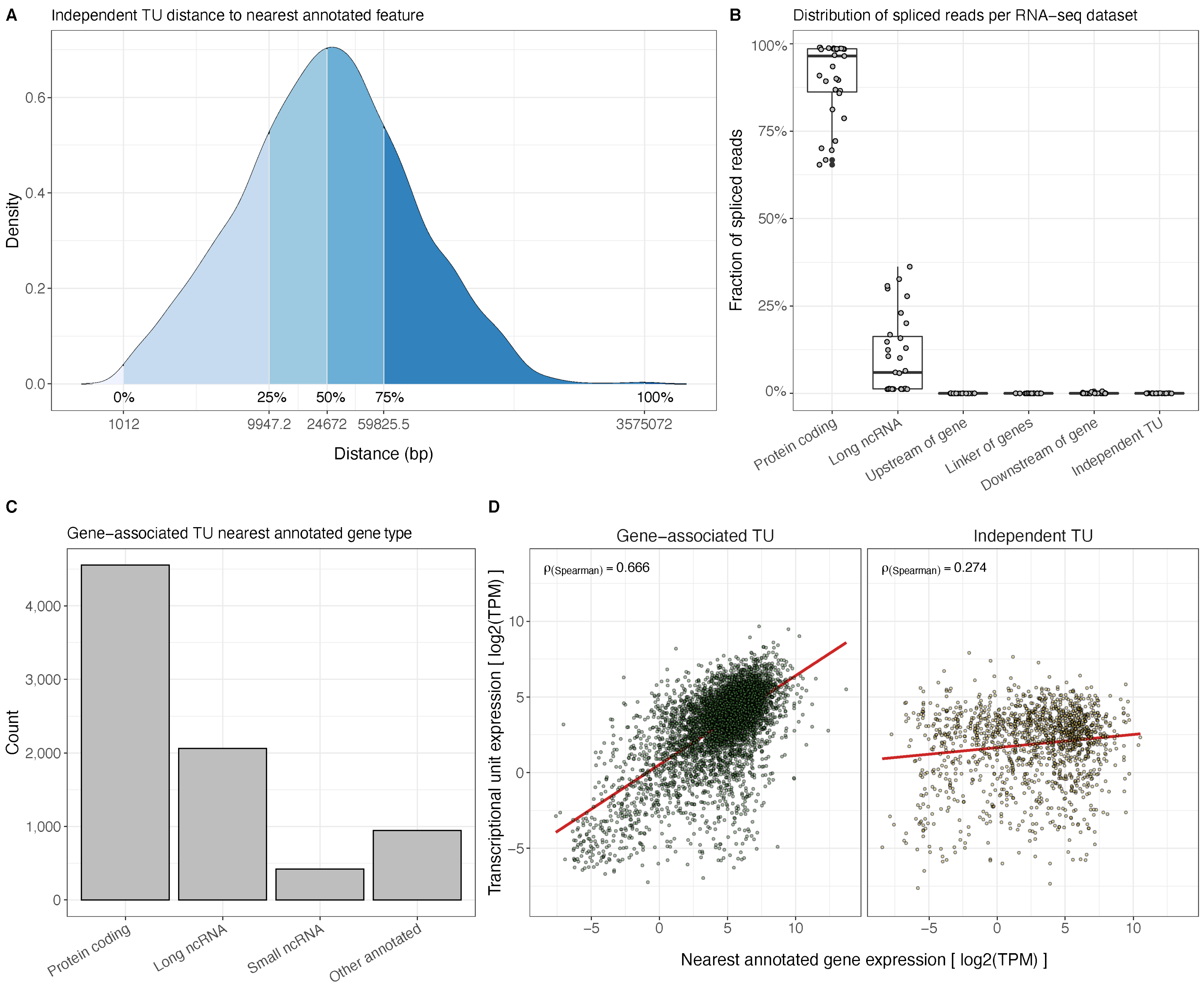

Figure S1. Summary of characteristics of gene-associated and independent TUs. A) Distribution of genomic distances between independent TUs and the nearest annotated gene. B) Distribution of proportions of spliced reads among annotated features and intergenic TUs; each data point represents an RNA-seq dataset included in this study. C) Gene-associated TUs nearest annotated gene type; D) Gene-associated and intergenic TUs expression against their nearest annotated gene expression.

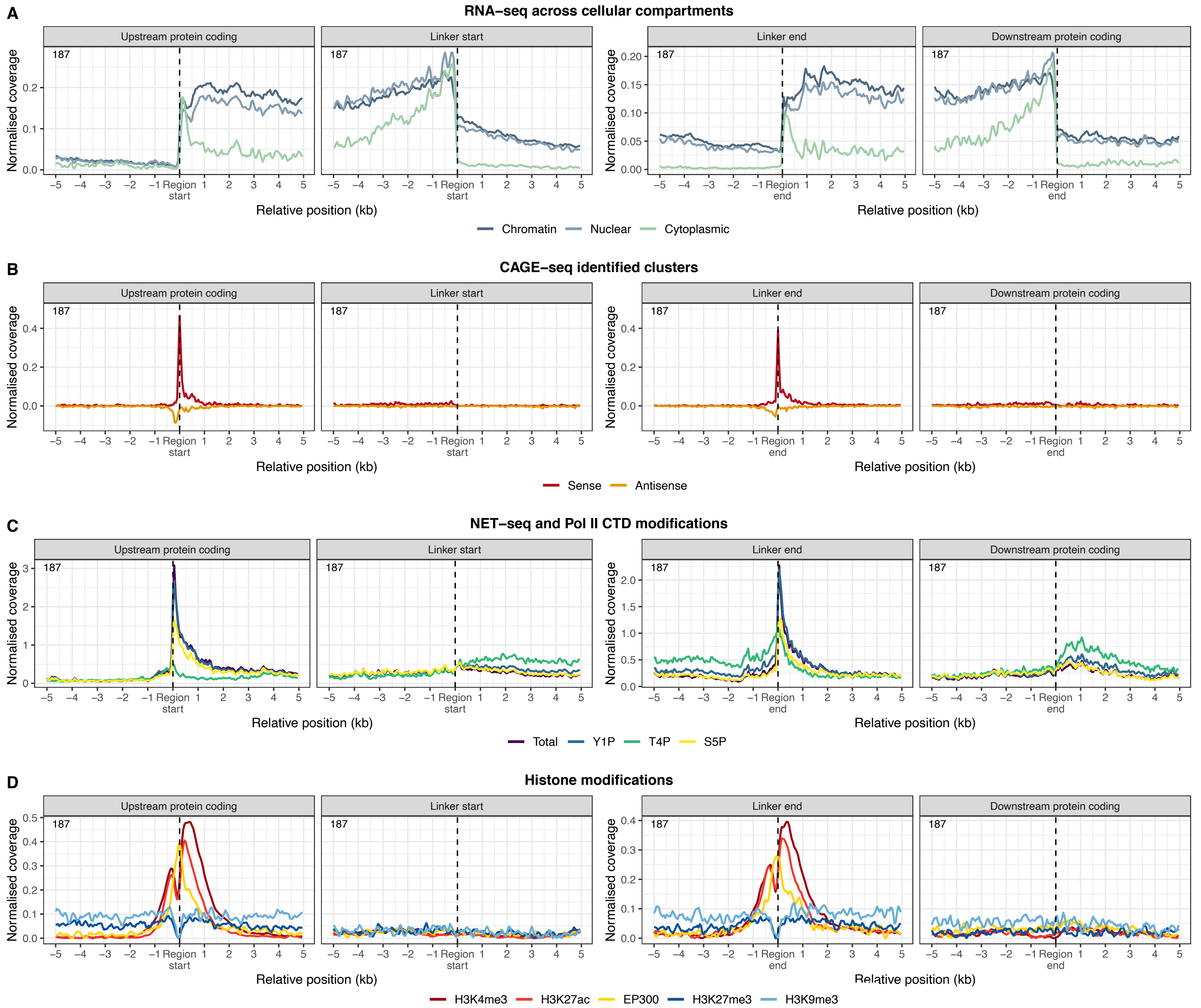

Figure S2. Meta-profiles of transcriptional measurements around LoGs. Meta-profiles of transcriptional measurements plotted relative to the start and end positions of LoGs and their associated protein-coding genes start positions. A) RNA-seq measurements in different subcellular compartments; B) CAGE-seq measurements in the sense and antisense strands; C) NET-seq measurements for different Pol II CTD modifications; D) ChIP-seq measurements for histone marks and EP300 occupancies associated transcriptional activities.

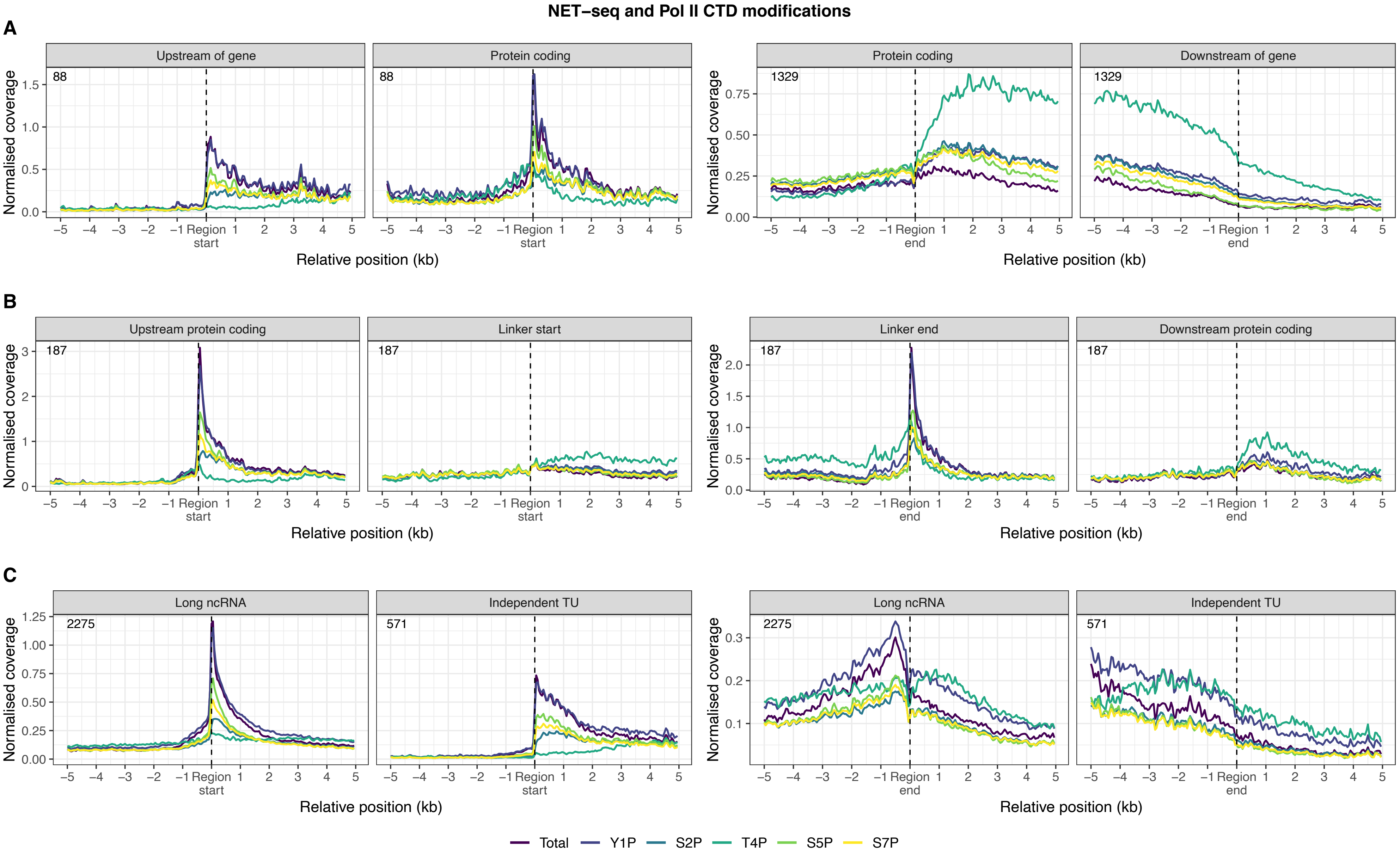

Figure S3. NET-seq meta-profiles for different Pol II CTD modifications around TUs. RNA polymerase II and its CTD modifications (NET-seq) occupancy profiles across: A) UoG regions and connected protein-coding genes start positions (left) and protein-coding genes and connected DoG regions end positions (right); B) upstream gene and LoG start (left) and LoG and downstream gene end (right) positions; C) long non-coding RNA genes and independent transcriptional units start (left) and end (right) positions.

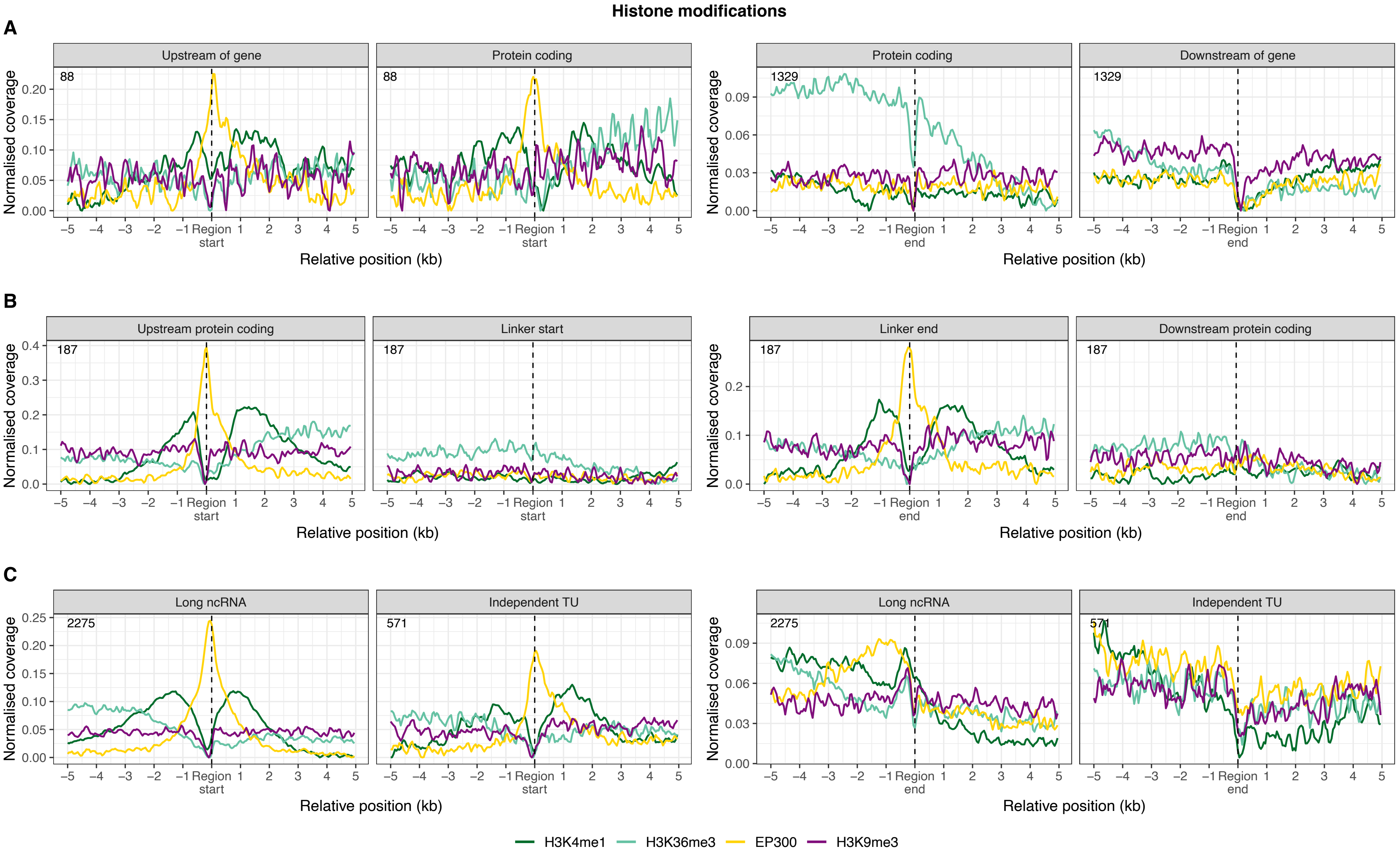

Figure S4. Enhancer epigenetic signature profiles. Enhancer-associated histone marks profiles across: A) UoG regions and connected protein-coding genes start positions (left) and protein-coding genes and connected DoG regions end positions (right); B) upstream gene and LoG start (left) and LoG and downstream gene end (right) positions; C) long non-coding RNA genes and independent transcriptional units start (left) and end (right) positions.

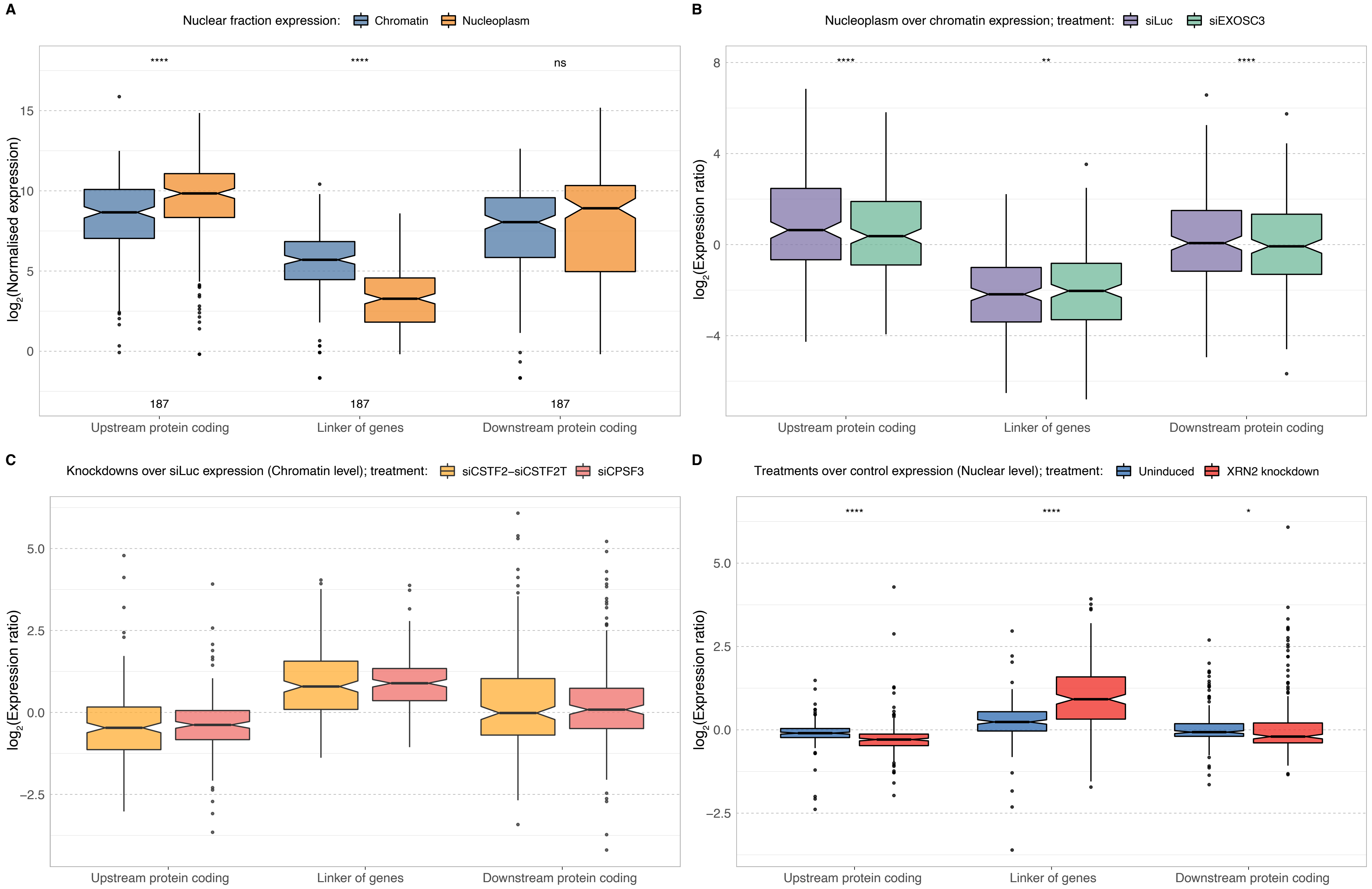

Figure S5. Impact of nuclease-depletion on gene-associated TU expression. A) Expression levels of protein-coding genes and linker of genes in the chromatin and nucleoplasm fractions. B) Relative nucleoplasmic-to-chromatin expression levels in response to EXOSC3 knockdown and control siLuc treatments. C) Expression levels in CSTF2+CSTF2T and CPSF3 knockdowns relative to control in the chromatin fraction. D) Expression levels in XRN2 knockdown (via activation of auxin-inducible degron system) and basal (uninduced; minus auxin) treatments relative to unmodified XRN2 control in the nuclear fraction. P values were calculated using the two-sided Wilcoxon rank sum test, with asterisks indicating statistical significance at the following thresholds: ns ($p > 0.05$); * ($p <= 0.05$); ** ($p <= 0.01$); *** ($p <= 0.001$); **** ($p <= 0.0001$).

Sample	Accession(s)	Experiment(s)	Total number of reads	Uniquely mapped	Uniquely mapped (%)	Mapped to multiple loci	Mapped to multiple loci (%)	Mapped to too many loci	Mapped to too many loci (%)
RNAseq_Chromatin-CPE_Untreated_HEK293_1_SE	GSE66478	SRR1824495	67018006	56893330	84.89%	784449	1.17%	9340227	13.94%
RNAseq_Chromatin-CPE_Untreated_HEK293_2_SE	GSE66478	SRR1824496	97732802	73403858	75.11%	977128	1.00%	23351816	23.89%
RNAseq_Chromatin-CPE_Untreated_HEK293_3_SE	GSE66478	SRR1824497	60382765	49709635	82.32%	661213	1.10%	10011917	16.58%
RNAseq_Chromatin_Untreated_HeLa_1_PE	GSE81662	SRR4436507	57683347	53814788	93.29%	1394394	2.42%	2474165	4.29%
RNAseq_Chromatin_Untreated_K562_1_PE	GSE90238	ENCFF000HGG,ENCFF000HHF	124316758	110933705	89.23%	7473665	6.02%	5909388	4.75%
RNAseq_Chromatin_Untreated_K562_2_PE	GSE90238	ENCFF000HGU,ENCFF000HHH	111260075	95947813	86.24%	8372862	7.52%	6939400	6.24%
RNAseq_Chromatin_siLuc_HeLa_1_PE	GSE81662	SRR3547470	22880833	19572835	85.54%	1997904	8.73%	1310094	5.73%
RNAseq_Chromatin_siLuc_HeLa_2_PE	GSE81662	SRR3547471	21824928	18668270	85.54%	1901880	8.71%	1254778	5.75%
RNAseq_Chromatin_siLuc_HeLa_3_PE	GSE81662	SRR3929125	19151960	17706267	92.45%	618639	3.23%	827054	4.32%
RNAseq_Chromatin_siLuc_HeLa_4_PE	GSE81662	SRR3929126	18377675	16995675	92.48%	583040	3.17%	798960	4.35%
RNAseq_Cytoplasm-pAm_Untreated_HepG2_1_PE	GSE90256	ENCFF000FWM,ENCFF000FWJ	24703857	15866682	64.23%	4027865	16.30%	4809310	19.47%
RNAseq_Cytoplasm-pAm_Untreated_HepG2_2_PE	GSE90256	ENCFF000FWL,ENCFF000FXG	21046274	13283451	63.12%	3687046	17.51%	4075777	19.37%
RNAseq_Cytoplasm-pAm_Untreated_K562_1_PE	GSE90249	ENCFF000HHN,ENCFF000HIK	10406483	5913424	56.82%	2796499	26.88%	1696560	16.30%
RNAseq_Cytoplasm-pAm_Untreated_K562_2_PE	GSE90249	ENCFF000HIQ,ENCFF000HIR	17124434	10005025	58.43%	4564777	26.65%	2554632	14.92%
RNAseq_Cytoplasm-pAp_Untreated_HepG2_1_PE	GSE90230	ENCFF000FXU,ENCFF000FYF	110688803	93093738	84.10%	5443069	4.92%	12151996	10.98%
RNAseq_Cytoplasm-pAp_Untreated_HepG2_2_PE	GSE90230	ENCFF000FYG,ENCFF000FYH	110050198	93706263	85.15%	4413974	4.01%	11929961	10.84%
RNAseq_Cytoplasm-pAp_Untreated_K562_1_PE	GSE90220	ENCFF000HJP,ENCFF000HJW	121945884	101017962	82.84%	5941489	4.87%	14986433	12.29%
RNAseq_Cytoplasm-pAp_Untreated_K562_2_PE	GSE90220	ENCFF000HJF,ENCFF000HJX	86284589	71792916	83.20%	5191582	6.02%	9300091	10.78%
RNAseq_Cytoplasm_WildType_HeLa_1_SE	E-MTAB-6204	ERS2004173	13975867	10981193	78.57%	92243	0.66%	2902431	20.77%
RNAseq_Cytoplasm_WildType_HeLa_2_SE	E-MTAB-6204	ERS2004174	8699329	7037520	80.90%	44972	0.51%	1616837	18.59%
RNAseq_Cytoplasm_WildType_HeLa_3_SE	E-MTAB-6204	ERS2004176	8926496	6820603	76.41%	68619	0.77%	2037274	22.82%
RNAseq_Nuclear-SNE_Untreated_HEK293_1_SE	GSE66478	SRR1824492	64342235	50343100	78.24%	492019	0.77%	13507116	20.99%
RNAseq_Nuclear-SNE_Untreated_HEK293_2_SE	GSE66478	SRR1824493	73083174	57527716	78.72%	588095	0.80%	14967363	20.48%
RNAseq_Nuclear-SNE_Untreated_HEK293_3_SE	GSE66478	SRR1824494	61119415	47486662	77.69%	477920	0.79%	13154833	21.52%
RNAseq_Nuclear-pAm_Untreated_HepG2_1_PE	GSE90248	ENCFF000FYM,ENCFF000FZR	93117065	80402123	86.35%	4359676	4.68%	8355266	8.97%
RNAseq_Nuclear-pAm_Untreated_HepG2_2_PE	GSE90248	ENCFF000FZL,ENCFF000FZS	106637948	89669476	84.09%	7156187	6.71%	9812285	9.20%
RNAseq_Nuclear-pAm_Untreated_K562_1_PE	GSE90250	ENCFF000HML,ENCFF000HNO	85809729	69869715	81.42%	4763917	5.56%	11176097	13.02%
RNAseq_Nuclear-pAm_Untreated_K562_2_PE	GSE90250	ENCFF000HMQ,ENCFF000HNP	78734856	64562145	82.00%	4037178	5.13%	10135533	12.87%
RNAseq_Nuclear-pAp_Untreated_HepG2_1_PE	GSE90228	ENCFF000GAF,ENCFF000GAT	100601844	86022775	85.51%	7359134	7.31%	7219935	7.18%
RNAseq_Nuclear-pAp_Untreated_HepG2_2_PE	GSE90228	ENCFF000GAH,ENCFF000GAS	83769437	69968863	83.53%	7510525	8.96%	6290049	7.51%
RNAseq_Nuclear-pAp_Untreated_K562_1_PE	GSE90236	ENCFF000HOC,ENCFF000HOE	114767203	99477619	86.68%	6536405	5.69%	8753179	7.63%
RNAseq_Nuclear-pAp_Untreated_K562_2_PE	GSE90236	ENCFF000HOD,ENCFF000HOQ	104460704	89483266	85.66%	7509727	7.19%	7467711	7.15%
RNAseq_Nuclear_Unmodified-XRN2_HCT116_1_SE	GSE109003	SRR6456886	46215564	40593226	87.83%	926141	2.01%	4696197	10.16%
RNAseq_Nuclear_Unmodified-XRN2_HCT116_2_SE	GSE109003	SRR6456889	32445099	28242564	87.05%	648955	2.00%	3553580	10.95%
RNAseq_Nuclear_WildType_HeLa_1_SE	E-MTAB-6204	ERS2004178	12322329	11601397	94.15%	105135	0.85%	615797	5.00%
RNAseq_Nuclear_WildType_HeLa_2_SE	E-MTAB-6204	ERS2004175	22482025	21203317	94.31%	180331	0.80%	1098377	4.89%
RNAseq_Nuclear_WildType_HeLa_3_SE	E-MTAB-6204	ERS2004177	23121311	21675348	93.75%	179337	0.77%	1266626	5.48%
RNAseq_Nucleoplasm-pAm_Untreated_HeLa_1_PE	GSE81662	SRR4436503	41600932	28960065	69.61%	2045296	4.92%	10595571	25.47%
RNAseq_Nucleoplasm-pAm_Untreated_HeLa_2_PE	GSE81662	SRR4436505	34695035	24609961	70.93%	1138317	3.28%	8946757	25.79%
RNAseq_Nucleoplasm-pAm_siLuc_HeLa_1_PE	GSE81662	SRR3547464	12762866	8686383	68.06%	1244036	9.75%	2832447	22.19%
RNAseq_Nucleoplasm-pAm_siLuc_HeLa_2_PE	GSE81662	SRR3547465	12938480	8516881	65.83%	1639405	12.67%	2782194	21.50%
RNAseq_Nucleoplasm-pAp_Untreated_HeLa_1_PE	GSE81662	SRR4436502	45205980	40606246	89.82%	768629	1.71%	3831105	8.47%
RNAseq_Nucleoplasm-pAp_Untreated_HeLa_2_PE	GSE81662	SRR4436504	46383260	41683722	89.87%	760050	1.64%	3939488	8.49%
RNAseq_Nucleoplasm-pAp_siLuc_HeLa_1_PE	GSE81662	SRR3547462	22247906	18907927	84.99%	1199882	5.39%	2140097	9.62%
RNAseq_Nucleoplasm-pAp_siLuc_HeLa_2_PE	GSE81662	SRR3547463	22262149	18547698	83.31%	1587723	7.14%	2126728	9.55%
RNAseq_Nucleoplasm_Untreated_HeLa_1_PE	GSE81662	SRR4436506	45341637	35408400	78.09%	1468785	3.24%	8464452	18.67%
RNAseq_Nucleoplasm_siLuc_HeLa_1_PE	GSE81662	SRR3547466	12213270	8716406	71.37%	642318	5.26%	2854546	23.37%
RNAseq_Nucleoplasm_siLuc_HeLa_2_PE	GSE81662	SRR3547467	11846173	8521493	71.93%	619063	5.23%	2705617	22.84%
RNAseq_Nucleoplasm_siLuc_HeLa_3_PE	GSE81662	SRR3929121	22929956	18362200	80.08%	621505	2.71%	3946251	17.21%
RNAseq_Nucleoplasm_siLuc_HeLa_4_PE	GSE81662	SRR3929122	22134437	17694119	79.94%	577741	2.61%	3862577	17.45%
RNAseq_PROseq_Chromatin_Bcells_1_SE	GSE39878	SRR1027467	221316904	208473163	94.20%	4864467	2.19%	7979274	3.61%
RNAseq_PROseq_Chromatin_Bcells_2_SE	GSE39878	SRR1027468	221771429	212259318	95.71%	1840115	0.83%	7671996	3.46%

Table S1. Annotation datasets. Accession numbers and mapping metrics.

Sample	Accession(s)	Experiment(s)	Total number of reads	Uniquely mapped	Uniquely mapped (%)	Mapped to multiple loci	Mapped to multiple loci (%)	Mapped to too many loci	Mapped to too many loci (%)
RNAseq_Chromatin_Untreated_HeLa_1_PE	GSE81662	SRR4436507	57683347	53814788	93.29%	1394394	2.42%	2474165	4.29%
RNAseq_Nucleoplasm_Untreated_HeLa_1_PE	GSE81662	SRR4436506	45341637	35408400	78.09%	1468785	3.24%	8464452	18.67%
RNAseq_Chromatin_Untreated_HeLa_1_PE	GSE60358	SRR1736053	38516999	31198238	81.00%	4256800	11.05%	3061961	7.95%
RNAseq_Chromatin_Untreated_HeLa_2_PE	GSE60358	SRR1736054	36266022	25372404	69.96%	5713292	15.76%	5180326	14.28%
RNAseq_Chromatin_siLuc_HeLa_1_PE	GSE60358	SRR1544612	27431795	22957068	83.69%	3014580	10.99%	1460147	5.32%
RNAseq_Chromatin_siCPSF3_HeLa_1_PE	GSE60358	SRR1544613	33261229	28254297	84.95%	3263432	9.81%	1743500	5.24%
RNAseq_Chromatin_siCSTF2-siCSTF2T_HeLa_1_PE	GSE60358	SRR1544614	26799776	21294949	79.46%	3191293	11.91%	2313534	8.63%
RNAseq_Chromatin_siCSTF2T_HeLa_1_PE	GSE60358	SRR1736056	20477740	15906615	77.68%	2453075	11.98%	2118050	10.34%
RNAseq_Chromatin_siCSTF2_HeLa_1_PE	GSE60358	SRR1736055	27256983	22432924	82.30%	3130844	11.49%	1693215	6.21%
RNAseq_Nuclear_Unmodified-XRN2_HCT116_1_SE	GSE109003	SRR6456886	46215564	40593226	87.83%	926141	2.01%	4696197	10.16%
RNAseq_Nuclear_Unmodified-XRN2_HCT116_2_SE	GSE109003	SRR6456889	32445099	28242564	87.05%	648955	2.00%	3553580	10.95%
RNAseq_Nuclear_XRN2-AID-mAuxin_HCT116_1_SE	GSE109003	SRR6456887	37943822	32516681	85.70%	980582	2.58%	4446559	11.72%
RNAseq_Nuclear_XRN2-AID-mAuxin_HCT116_2_SE	GSE109003	SRR6456890	48093657	42252039	87.85%	1024331	2.13%	4817287	10.02%
RNAseq_Nuclear_XRN2-AID-pAuxin_HCT116_1_SE	GSE109003	SRR6456888	46494173	41120530	88.44%	852857	1.84%	4520786	9.72%
RNAseq_Nuclear_XRN2-AID-pAuxin_HCT116_2_SE	GSE109003	SRR6456891	24792285	21231296	85.64%	499495	2.01%	3061494	12.35%
NETseq_mNETseq_Untreated_HeLa_1_PE	GSE81662	SRR4436478	24882369	20142589	80.95%	2060884	8.28%	2678896	10.77%
NETseq_mNETseq_Untreated_HeLa_2_PE	GSE81662	SRR4436479	24844889	20113619	80.96%	2055232	8.27%	2676038	10.77%
NETseq_mNETseq_Untreated-S2P_HeLa_1_PE	GSE81662	SRR3547442	25126117	20760264	82.62%	1830039	7.29%	2535814	10.09%
NETseq_mNETseq_Untreated-S2P_HeLa_2_PE	GSE81662	SRR3547443	25242424	20856406	82.62%	1839025	7.29%	2546993	10.09%
NETseq_mNETseq_Untreated-S5P_HeLa_1_PE	GSE81662	SRR3547438	23568445	19806348	84.04%	1388183	5.89%	2373914	10.07%
NETseq_mNETseq_Untreated-S5P_HeLa_2_PE	GSE81662	SRR3547439	23636831	19832005	83.90%	1436131	6.08%	2368695	10.02%
NETseq_mNETseq_Untreated-S7P_HeLa_1_PE	GSE81662	SRR3547428	60551522	50616390	83.59%	4197154	6.93%	5737978	9.48%
NETseq_mNETseq_Untreated-S7P_HeLa_2_PE	GSE81662	SRR3547429	60798794	50751517	83.47%	4321652	7.11%	5725625	9.42%
NETseq_mNETseq_Untreated-S7P_HeLa_3_PE	GSE81662	SRR4436476	32336002	25328193	78.33%	3262181	10.09%	3745628	11.58%
NETseq_mNETseq_Untreated-S7P_HeLa_4_PE	GSE81662	SRR4436477	32283735	25289904	78.34%	3258769	10.09%	3735062	11.57%
NETseq_mNETseq_Untreated-T4P_HeLa_1_PE	GSE81662	SRR3547426	35502828	28932090	81.49%	2900842	8.17%	3669896	10.34%
NETseq_mNETseq_Untreated-T4P_HeLa_2_PE	GSE81662	SRR3547427	35611824	29046567	81.56%	2876901	8.08%	3688356	10.36%
NETseq_mNETseq_Untreated-Y1P_HeLa_1_PE	GSE81662	SRR3547424	63850355	53965445	84.52%	3813126	5.97%	6071784	9.51%
NETseq_mNETseq_Untreated-Y1P_HeLa_2_PE	GSE81662	SRR3547425	64155974	54149654	84.40%	3943183	6.15%	6063137	9.45%
RNAseq_Chromatin_siLuc_HeLa_1_PE	GSE81662	SRR3547470	22880833	19572835	85.54%	1997904	8.73%	1310094	5.73%
RNAseq_Chromatin_siLuc_HeLa_2_PE	GSE81662	SRR3547471	21824928	18668270	85.54%	1901880	8.71%	1254778	5.75%
RNAseq_Chromatin_siLuc_HeLa_3_PE	GSE81662	SRR3929125	19151960	17706267	92.45%	618639	3.23%	827054	4.32%
RNAseq_Chromatin_siLuc_HeLa_4_PE	GSE81662	SRR3929126	18377675	16995675	92.48%	583040	3.17%	798960	4.35%
RNAseq_Nucleoplasm_siLuc_HeLa_1_PE	GSE81662	SRR3547466	12213270	8716406	71.37%	642318	5.26%	2854546	23.37%
RNAseq_Nucleoplasm_siLuc_HeLa_2_PE	GSE81662	SRR3547467	11846173	8521493	71.93%	619063	5.23%	2705617	22.84%
RNAseq_Nucleoplasm_siLuc_HeLa_3_PE	GSE81662	SRR3929121	22929956	18362200	80.08%	621505	2.71%	3946251	17.21%
RNAseq_Nucleoplasm_siLuc_HeLa_4_PE	GSE81662	SRR3929122	22134437	17694119	79.94%	577741	2.61%	3862577	17.45%
RNAseq_Chromatin_siEXOC3_HeLa_1_PE	GSE81662	SRR3547472	35455828	29388055	82.89%	3748736	10.57%	2319037	6.54%
RNAseq_Chromatin_siEXOC3_HeLa_2_PE	GSE81662	SRR3547473	34229538	28373374	82.89%	3612923	10.56%	2243241	6.55%
RNAseq_Chromatin_siEXOC3_HeLa_3_PE	GSE81662	SRR3929127	22360783	20485434	91.61%	669330	3.00%	1206019	5.39%
RNAseq_Chromatin_siEXOC3_HeLa_4_PE	GSE81662	SRR3929128	21545197	19745607	91.65%	635030	2.94%	1164560	5.41%
RNAseq_Nucleoplasm_siEXOC3_HeLa_1_PE	GSE81662	SRR3547468	13783740	10055143	72.95%	675706	4.90%	3052891	22.15%
RNAseq_Nucleoplasm_siEXOC3_HeLa_2_PE	GSE81662	SRR3547469	13233330	9705351	73.34%	638879	4.83%	2889100	21.83%
RNAseq_Nucleoplasm_siEXOC3_HeLa_3_PE	GSE81662	SRR3929123	13812366	11222534	81.25%	370511	2.68%	2219321	16.07%
RNAseq_Nucleoplasm_siEXOC3_HeLa_4_PE	GSE81662	SRR3929124	13315969	10831672	81.34%	349505	2.63%	2134792	16.03%

Table S2. Validation datasets. Accession numbers and mapping metrics.

Cell	Target	Accession(s)	Experiment(s)	Format	Type	Replicate(s)
HeLa-S3	H3K27me3	GSE29611	ENCFF614HNF	bigWig	fold change over control	1,2
HeLa-S3	H3K27me3	GSE29611	ENCFF772IJJ	bigWig	fold change over control	1,2
HeLa-S3	H3K36me3	GSE29611	ENCFF521FCP	bigWig	fold change over control	1,2
HeLa-S3	H3K79me2	GSE29611	ENCFF303MHF	bigWig	fold change over control	1,2
HeLa-S3	H4K20me1	GSE29611	ENCFF324PJA	bigWig	fold change over control	1,2
HeLa-S3	H3K27ac	GSE29611	ENCFF194XTD	bigWig	fold change over control	1,2
HeLa-S3	H3K4me1	GSE29611	ENCFF430ZMK	bigWig	fold change over control	1,2
HeLa-S3	H3K4me2	GSE29611	ENCFF752UST	bigWig	fold change over control	1,2
HeLa-S3	H3K4me3	GSE29611	ENCFF913SUG	bigWig	fold change over control	1,2
HeLa-S3	H3K4me3	GSE29611	ENCFF509ECR	bigWig	fold change over control	1,2
HeLa-S3	H3K4me3	GSE29611	ENCFF489CIY	bigWig	fold change over control	1,2
HeLa-S3	H3K9me3	GSE29611	ENCFF891XLY	bigWig	fold change over control	1,2
HeLa-S3	H3K9ac	GSE29611	ENCFF431ISH	bigWig	fold change over control	1,2
HeLa-S3	H2A.Z	GSE29611	ENCFF642VOB	bigWig	fold change over control	1,2
HepG2	H3K27me3	GSE29611	ENCFF419FUZ	bigWig	fold change over control	1,2
HepG2	H3K27me3	GSE29611	ENCFF598TWA	bigWig	fold change over control	1,2
HepG2	H3K36me3	GSE29611	ENCFF104ECN	bigWig	fold change over control	1,2
HepG2	H3K36me3	GSE29611	ENCFF550PXE	bigWig	fold change over control	1,2
HepG2	H4K20me1	GSE29611	ENCFF554JDF	bigWig	fold change over control	1,2
HepG2	H3K27ac	GSE29611	ENCFF764VYK	bigWig	fold change over control	1,2
HepG2	H3K4me1	GSE29611	ENCFF058GCZ	bigWig	fold change over control	1,2
HepG2	H3K4me2	GSE29611	ENCFF109QAV	bigWig	fold change over control	1,2
HepG2	H3K4me3	GSE29611	ENCFF777EVS	bigWig	fold change over control	1,2
HepG2	H3K4me3	GSE29611	ENCFF736LHE	bigWig	fold change over control	1,2
HepG2	H3K4me3	GSE29611	ENCFF746CXV	bigWig	fold change over control	1,2
HepG2	H3K9me3	GSE29611	ENCFF485BCI	bigWig	fold change over control	1,2
HepG2	H3K9ac	GSE29611	ENCFF053ROV	bigWig	fold change over control	1,2
K562	H3K27me3	GSE29611	ENCFF914VFE	bigWig	fold change over control	1,2
K562	H3K27me3	GSE29611	ENCFF928NWQ	bigWig	fold change over control	1,2
K562	H3K36me3	GSE29611	ENCFF440XMD	bigWig	fold change over control	1,2
K562	H3K36me3	GSE29611	ENCFF954JHK	bigWig	fold change over control	1,2
K562	H3K79me2	GSE29611	ENCFF901YVS	bigWig	fold change over control	1,2
K562	H3K27ac	GSE29611	ENCFF779QTH	bigWig	fold change over control	1,2
K562	H3K4me1	GSE29611	ENCFF761XBZ	bigWig	fold change over control	1,2
K562	H3K4me2	GSE29611	ENCFF491AUC	bigWig	fold change over control	1,2
K562	H3K4me3	GSE29611	ENCFF525ZRM	bigWig	fold change over control	1,2
K562	H3K4me3	GSE29611	ENCFF847JMY	bigWig	fold change over control	1,2
K562	H3K4me3	GSE29611	ENCFF814IYI	bigWig	fold change over control	1,2
K562	H3K4me3	GSE29611	ENCFF712XRE	bigWig	fold change over control	1,2
K562	H3K9me3	GSE29611	ENCFF812HRW	bigWig	fold change over control	1,2
K562	H3K9ac	GSE29611	ENCFF911SSE	bigWig	fold change over control	1,2
K562	H3K9ac	GSE29611	ENCFF937QUK	bigWig	fold change over control	1,2
K562	H3K9ac	GSE29611	ENCFF115LJM	bigWig	fold change over control	1,2
K562	H2A.Z	GSE29611	ENCFF494WCA	bigWig	fold change over control	1,2

Table S3. Histone modifications datasets. Accession numbers and file type details.

Independent transcriptional units (n = 1826) overlaps with publicly available gene databases

	Percentage of independent TU minimum required overlap									
**Dataset**	**10%**	**20%**	**30%**	**40%**	**50%**	**60%**	**70%**	**80%**	**90%**	**100%**
NONCODE (n = 268524)	994	948	893	843	790	745	704	648	583	354
CHESS (n = 4863091)	498	470	458	436	409	383	360	339	305	210
RefSeq (n = 994884)	1034	1025	1023	1020	1012	1004	997	988	979	959

Table S4. Independent TUs comparison with known databases. The numbers of overlapping independent TU regions, at different coverage fractions, are reported for NONCODE, CHESS and RefSeq annotation references. Overlaps were calculated using BEDtools intersect function.
