# Supplementary material for: Intergenic RNA mainly derives from nascent transcripts of known genes
Source: Genome Biol. 2021 May 5;22:136. doi: 10.1186/s13059-021-02350-x (PMC8097831; doi:10.1186/s13059-021-02350-x)
Supplement: Supplementary file 2 — Additional file 2. Review history. [file 13059_2021_2350_MOESM2_ESM.docx]

**1^st^ round**

**Reviewer 1**

In the manuscript titled "Intergenic RNA mainly derives from nascent transcripts of known genes" the authors provide compelling reasons that suggest a non-stochastic nature for the RNA Pol II binding of upstream of gene transcripts and the readthrough nature of downstream of gene transcripts. Additionally, the authors identify 1826 independent transcriptional units (TUs) as likely candidates for being annotated as lncRNA or mRNA. The study builds on the previous works and provides further evidence through comprehensive computational analysis of multiple genomic assays (RNAseq, CAGE peak profiles, ChIP-seq, NET-seq) and several subcellular fractions to investigate the properties of intergenic transcription. The authors put together a protocol that can be used by other studies that seek to refine and augment eukaryotic gene annotations.

However, several problems with the manuscript require additional work to correct inconsistencies, elucidate several ambiguities and provide additional justification to their findings as detailed below.

Major revisions:

1. In the abstract the authors state that they built an atlas of intergenic transcription. This atlas should be provided with the submission, or a link to the atlas should be mentioned in the manuscript.

2. Throughout the manuscript the authors make multiple mentions of the custom wrappers and integrated protocol that they designed and used for their analysis. While the methods section and figure 1 do a good job of explaining the parameters and some steps in the protocol, the code should be made publicly available preferably under an open-source license.

3. Authors should search their extended gene coordinates and novel independent TUs in other publicly available gene catalogs (such as RefSeq, CHESS, NONCODE, etc.). Independent TUs might have already been classified as such in those sources, providing further evidence for some of the claims the authors make.

4. The authors claim to have classified "7,411 TUs covering ~5.6% of the human genome", and augment the statement with Figure 2C and 2D. However, by roughly estimating mean TU lengths from Figure 2D and computing the total number of bases contained (assuming no overlaps or alternative splicing) the number ends up closer to ~50 Mb which is far less that 5.6% (~170 Mb). This statement should be corrected or made more clear.

5. Authors used QoRT to quantify expression of GENCODE-annotated features. Later in the Methods, the authors state that the expression was reassessed post-assembly. How was the expression re-assessed? Why were expression estimates collected by an independent method, if StringTie performs quantification by default with respect to the assembled features?

6. Why were TUs smaller than 1kb discarded? The authors should comment on this as this seems like a rather strict requirement.

Minor revisions

1. Gene-associated TUs were selected as being within 5Kb of the annotated loci. The authors do not make it clear in the manuscript whether contiguous coverage was required to make the association. If not, the authors should clarify how such TUs were differentiated from independent TUs, since GENCODE v29 contains ~2900 lncRNA and protein-coding genes within such limits of proximity to one another.

2. While the decision to remove multi-mapping reads from the analysis certainly benefits the specificity of the results, authors should clarify whether alternative scaffolds of the GRCh38 were included in the analysis. If so, perhaps the removal of ALT scaffolds from the STAR and Bowtie indices as well as GENCODE annotation would allow identification of additional TUs?

3. Several citations might be warranted as described below:

a. Suggested citation for the following statement: "Despite such endeavours however, a marked proportion of RNA-seq reads from human cells still map to unannotated, ostensibly intergenic portions of the human genome."

b. The following publication seems relevant to this study as it describes similar results: Van Bakel, Harm, et al. "Most "dark matter" transcripts are associated with known genes." PLoS biology 8.5 (2010).

c. Suggested citation for p.5 l.46 "This observation implies that although Pol II is poised to terminate after encountering the canonical TES, actual Pol II detachment might occur several kilobases downstream.". This has been previously stated in Lian, Zheng, et al. "A genomic analysis of RNA polymerase II modification and chromatin architecture related to 3′ end RNA polyadenylation." Genome research 18.8 (2008): 1224-1237.

4. The publicly available data is heterogeneous in terms of library prep (paired/single, 50bp-100bp long reads). Authors should specify the minimum read length retained after trimming by TrimGallore and the minimum alignment length for STAR/Bowtie2. The discussion could benefit from a small mention of the implications of shorter read length.

7. It could be informative to include the number of spliced reads for the UoGs, LoGs and DoGs. If spliced reads where detected in those TUs, could that then be a sign of potentially functional transcription which should be annotated for those loci as opposed to being a readthrough event?

8. The use of TUs is not well defined. Does it refer to loci or transcripts? How many TUs are alternative isoforms within the same locus, especially within the independent TU set?

9. The authors state that "independent TUs appear to be bona fide lincRNAs that eluded reference annotation." However, in supplementary Figure 1B authors also report near complete absence of spliced reads in independent TUs in contrast to ~7-10% spliced reads in selected group of similarly sized lncRNA. Is there a suggested reason for the difference in splicing between the two groups?

Typos and ambiguities:

1. Supplementary Figure 1 A, Title - Independent TU distance to neatest annotated gene

2. P.5 l.45 - and causes a characteristic, NET-Seq

3. p.8 l.48 - remodelers that are able to to maintain an open

4. p.8 l.56 - by nuclear surveillance processes suggest their

5. p.8 l.60 - via the torpedo mechanism model, in which the exonuclease degrades the gene-associated RNA until it reaches the elongation complex so causing its termination

6. p.9 l.11 - to exert their functional role in trans

7. p.9 l.56 - Deduplicated unique alignments were parse using samtools

8. p.10 l.48 - and gapped alignments (i.e., reads encompassing known or putative splice junctions) we extracted based on their CIGAR information

9. p.11 l.37 - and several proteins knockdowns

10. p.11 l.44 - used to normalised the feature counts

11. p.11 l.44 - ggpubr - needs to be italicised for consistency

**Reviewer 2**

The study by Agostini et al attempts to stratify the vast amount of the long non-coding (lnc) RNA transcription in the human genome into two main classes - (1) lncRNAs associated with known genes and thus representing products of (by the authors definition) "fuzzy" transcription from those genes and (2) stand alone lncRNA units. To accomplish this task, the authors have analyzed a large amount of publicly available transcriptome and chromatin data.

Conceptual comments:

1. After reading the paper, I am confused about what the authors aim to accomplish and what their main conclusions are. Overall, the logic is very confusing in the text. For example, in terms of the conclusions, in the Introduction, they state that "We conclude that most of the unannotated intergenic RNAs are the consequence of non-productive transcription associated with known genes, which are rapidly removed through cellular quality control mechanisms." However, in Discussion's section "Functional consequences of non-canonical transcription on canonical genes" they describe in details potential functional consequences of the gene-associated transcription. Therefore, even the main conclusion from the paper is not exactly clear.

2. However, even more importantly, it is not quite clear what the authors are trying to achieve here. If the main goal is to characterize such features as association of the lncRNA transcription with protein-coding genes, chromatin- and nuclear-localization and their stability as well as some others, then all this has been already shown in multiple previous studies - see for example PMIDs 28017589, 27870833, 23006825, 26190259, 22955620 just to list a few. In this regard, the authors do not seem to provide novel insights.

3. On the other hand, if the authors are trying to use the association with genes as a mean to annotate the lncRNAs in terms of their biological relevance, then this approach is inherently flawed. First and foremost, association with known genes per se can not be used as the evidence of absence of biological function. For example, numerous studies have shown that intronic transcripts - the quintessential gene-associated transcripts - represent a rich source of potentially-functional RNA species as summarized in two recent reviews PMIDs 30391089 and 31668856.

4. If the authors are trying to provide some sort of a classification scheme to sort lncRNAs into potentially functional or not based on their association with genes, then the results presented are too preliminary and insufficient to address this. At the very least, the authors should provide functional validations of their findings to show that transcripts that are classified as non-functional in their studies indeed do not have functions and vice versa.

5. The validation work included in the manuscript is not clear. For example, why did the authors choose a separate cell line for the validation considering that lncRNA transcripts are well-known to be cell-type specific? What was the rationale for using HeLa for this purpose? Exactly what the authors were validating and how? All this is not clear.

Technical comments:

1. The authors provide multiple comparisons between chromatin, CAGE and RNAseq data. However, its not immediately clear if these comparisons are valid. Some of these datasets are based on a very broad numbers of cell types. However, it is a well-known fact that expression of lncRNAs is much more cell-type specific compared to the protein-coding mRNAs. Are the authors limiting their comparison to the same cell type or are they comparing bulk data? If the latter, the lncRNA-associated chromatin or CAGE signal can be easily lost.

2. The pipeline presented in Figure 1 describes only the basic analysis of the RNAseq data and does not show how the lncRNA transcripts were classified into the different classes. The authors cite custom pipelines in the text without providing any details and any rationale for choosing specific parameters for this classification.

Conclusion:

Overall, the manuscript offers no sufficiently-novel insights and contains too many flaws making it unsuitable for publication in the present form.

**Editors’ comments**

We rejected the manuscript at this stage, but the authors appealed, saying they could address the reviewers’ concerns, so we allowed a resubmission.

**Author response**

**Reviewer 1**

In the manuscript titled "Intergenic RNA mainly derives from nascent transcripts of known genes" the authors provide compelling reasons that suggest a non-stochastic nature for the RNA Pol II binding of upstream of gene transcripts and the readthrough nature of downstream of gene transcripts. Additionally, the authors identify 1826 independent transcriptional units (TUs) as likely candidates for being annotated as lncRNA or mRNA. The study builds on the previous works and provides further evidence through comprehensive computational analysis of multiple genomic assays (RNAseq, CAGE peak profiles, ChIP-seq, NET-seq) and several subcellular fractions to investigate the properties of intergenic transcription. The authors put together a protocol that can be used by other studies that seek to refine and augment eukaryotic gene annotations.

However, several problems with the manuscript require additional work to correct inconsistencies, elucidate several ambiguities and provide additional justification to their findings as detailed below.

Major revisions:

1. In the abstract the authors state that they built an atlas of intergenic transcription. This atlas should be provided with the submission, or a link to the atlas should be mentioned in the manuscript.

Response: We thank the reviewer for the advice. We have now included the annotation (BED) files (and links to open them in the UCSC Genome Browser) of the identified intergenic regions in the github repository (<https://github.com/luslab/IntergenicTranscriptionManuscript>).

2. Throughout the manuscript the authors make multiple mentions of the custom wrappers and integrated protocol that they designed and used for their analysis. While the methods section and figure 1 do a good job of explaining the parameters and some steps in the protocol, the code should be made publicly available preferably under an open-source license.

R: We have now provided a github repository to the code under a GPL3 open-source license (<https://github.com/luslab/IntergenicTranscriptionManuscript>).

3. Authors should search their extended gene coordinates and novel independent TUs in other publicly available gene catalogs (such as RefSeq, CHESS, NONCODE, etc.). Independent TUs might have already been classified as such in those sources, providing further evidence for some of the claims the authors make.

R: We thank the reviewer for the suggestion. We evaluated the overlaps of the independent TUs with the suggested gene catalogs. We found that 994 (54%), 498 (27%) and 1034 (57%) independent TUs overlap (at least 10% of the region) annotated features of CHESS, NONCODE and RefSeq, respectively. In addition, we found 373 (20%) independent TUs that we identified that have not been reported in any of the aforementioned databases, thus indicating that our results, on top of being supported by external sources, also expand the current catalogue of transcribed RNAs. We included supplementary table 2 that shows the intersection with the databases at different fractions of overlap between features.

4. The authors claim to have classified "7,411 TUs covering ~5.6% of the human genome", and augment the statement with Figure 2C and 2D. However, by roughly estimating mean TU lengths from Figure 2D and computing the total number of bases contained (assuming no overlaps or alternative splicing) the number ends up closer to ~50 Mb which is far less that 5.6% (~170 Mb). This statement should be corrected or made more clear.

R: Actually, this assumption is not as straightforward: the coverage cannot be extracted from figure 2C and 2D. First, what the reviewer refers to as the mean in the box plot is actually the median (we could add the mean as a dot on top of each boxplot, if this would clarify the point). Second, the coverage in 2E refers to all TUs, while 2C and 2D are filtered by expression in HeLa cells. Thus, the 5.6% is correct.

5. Authors used QoRT to quantify expression of GENCODE-annotated features. Later in the Methods, the authors state that the expression was reassessed post-assembly. How was the expression re-assessed? Why were expression estimates collected by an independent method, if StringTie performs quantification by default with respect to the assembled features?

R: QoRts was used only to estimate GENCODE features, and this is stated both in figure 1 and the Methods. After the assembly, the expression is re-assessed with a similar approach (i.e. countOverlapsUnion() function), because the data was loaded into R and, in order to re-run QoRTs, we would have had to export the features, run QoRTs and then import it back into R. StringTie performs the quantification per sample and, for each, reports a different set of features (i.e. some might have different lengths or be present in one but not in the other samples), and therefore not all ‘new’ features can be simultaneously quantified across all samples. We first merged the StringTie outputs within R and then and then re-assessed the features’ expression.

6. Why were TUs smaller than 1kb discarded? The authors should comment on this as this seems like a rather strict requirement.

R: We apologise if this created some confusion. We initially decided to discard <1 kb TUs because it was not our intention to redefine slightly shifted annotated gene boundaries. Although these can represent true signals, the low number of supporting reads observed in these small regions can be seen as a consequence of misannotation (gene-associated TUs) or misalignment (independent TUs). We included this explanation in the Methods.

Minor revisions

1. Gene-associated TUs were selected as being within 5Kb of the annotated loci. The authors do not make it clear in the manuscript whether contiguous coverage was required to make the association. If not, the authors should clarify how such TUs were differentiated from independent TUs, since GENCODE v29 contains ~2900 lncRNA and protein-coding genes within such limits of proximity to one another.

R: As stated in the manuscript, continuous coverage is the requirement for calling these features gene-associated: “The remaining are further divided into “geneassociated” and “independent” transcriptional units (“gene-associated TUs” and “independent TUs”, respectively) based on whether they originate from annotated genes, thus showing transcriptional continuity with the gene body, or from regions devoid of annotated features, and therefore they are considered independent events of transcription.” (page 10). Exactly for the issue raised in the second part of the comment, we excluded from the independent TUs those regions that were <10 kb from annotated genes, a threshold that is supported by figure S1A.

2. While the decision to remove multi-mapping reads from the analysis certainly benefits the specificity of the results, authors should clarify whether alternative scaffolds of the GRCh38 were included in the analysis. If so, perhaps the removal of ALT scaffolds from the STAR and Bowtie indices as well as GENCODE annotation would allow identification of additional TUs?

R: This is an intriguing possibility. However, it is not the purpose of this study to investigate the effect of the outcomes of these algorithms, which do not have contigsaware releases, on the annotation of unannotated features.

3. Several citations might be warranted as described below:

a. Suggested citation for the following statement: "Despite such endeavours however, a marked proportion of RNA-seq reads from human cells still map to unannotated, ostensibly intergenic portions of the human genome."

b. The following publication seems relevant to this study as it describes similar results: Van Bakel, Harm, et al. "Most "dark matter" transcripts are associated with known genes." PLoS biology 8.5 (2010).

c. Suggested citation for p.5 l.46 "This observation implies that although Pol II is poised to terminate after encountering the canonical TES, actual Pol II detachment might occur several kilobases downstream.". This has been previously stated in Lian, Zheng, et al. "A genomic analysis of RNA polymerase II modification and chromatin architecture related to 3′ end RNA polyadenylation." Genome research 18.8 (2008): 1224-1237.

R: As mentioned above, in van Bakel et al. (PLOS Biol., 2010), the authors explored transcribed unannotated transcripts by using tiling arrays and PolyA+ RNA-seq technologies, none of which is able to thoroughly annotate intergenic transcription. In our study, we take advantage of large amounts of nuclear and chromatin subcellular fractions and NET-seq data, which are much more powerful and comprehensive for the detection of short-lived long RNAs, such as the products of downstream-of-gene transcription. We added the suggested references and a comment on the previous work by van Bakel and colleagues in the Discussion.

4. The publicly available data is heterogeneous in terms of library prep (paired/single, 50bp-100bp long reads). Authors should specify the minimum read length retained after trimming by TrimGallore and the minimum alignment length for STAR/Bowtie2. The discussion could benefit from a small mention of the implications of shorter read length.

R: We stated in the Methods (page 9) that TrimGalore was run with default parameters and, as such, the default minimum read length retained is 20. Moreover, all STAR/Bowtie2 parameters used for the alignments are reported in the Methods; if not, their values fall back to the algorithms defaults. We believe discussing the implication of shorter reads length goes beyond the scope of this work, as it has already been covered in a number of studies (e.g. PMID: 25426137, 26100517, 24376861).

7. It could be informative to include the number of spliced reads for the UoGs, LoGs and DoGs. If spliced reads where detected in those TUs, could that then be a sign of potentially functional transcription which should be annotated for those loci as opposed to being a read-through event?

R: We thank the reviewer for the suggestion. We have now expanded the spliced reads analysis in Supplementary Figure 1B by including protein-coding genes and gene-associated TUs (UoGs, LoGs and DoGs). The latter are clearly depleted in splicing events, thus supporting the idea that these regions are not going to be part of the transcripts generated from the annotated gene body. Additionally, it has been shown that splicing is generally less conserved and less regulated for certain classes of RNAs (Krchňáková Z., et al., 2019), suggesting that splicing alone cannot be used as a definitive indicator of function.

8. The use of TUs is not well defined. Does it refer to loci or transcripts? How many TUs are alternative isoforms within the same locus, especially within the independent TU set?

R: The term TU refers to the transcriptional locus, and there can be only one TU per locus. The actual quantification of alternative isoforms would require a more sophisticated modelling, similar to DEXseq, which would take into account the unique non-overlapping fragments that can be found in each region. However, this approach takes advantage of pre-existing information on annotated exons to create isoforms in a combinatorial way. In our case, we do not have sub-regions within the TUs, and therefore we cannot model isoforms usage.

9. The authors state that "independent TUs appear to be bona fide lincRNAs that eluded reference annotation." However, in supplementary Figure 1B authors also report near complete absence of spliced reads in independent TUs in contrast to ~7- 10% spliced reads in selected group of similarly sized lncRNA. Is there a suggested reason for the difference in splicing between the two groups?

R: Not all the long ncRNAs are undergoing splicing (e.g. PMID: 26289325), such as “macro lncRNA” (e.g. PMID: 22617879) that are only recently receiving increasing attention. We cannot exclude the possibility that our independent TUs can be part of this group.

10. Typos and ambiguities:

10.1. Supplementary Figure 1 A, Title - Independent TU distance to neatest annotated

gene

10.2. P.5 l.45 - and causes a characteristic, NET-Seq

10.3. p.8 l.48 - remodelers that are able to to maintain an open

10.4. p.8 l.56 - by nuclear surveillance processes suggest their

10.5. p.8 l.60 - via the torpedo mechanism model, in which the exonuclease degrades

the gene-associated RNA until it reaches the elongation complex so causing its

termination

10.6. p.9 l.11 - to exert their functional role in trans

10.7. p.9 l.56 - Deduplicated unique alignments were parse using samtools

10.8. p.10 l.48 - and gapped alignments (i.e., reads encompassing known or putative

splice junctions) we extracted based on their CIGAR information

10.9. p.11 l.37 - and several proteins knockdowns

10.10. p.11 l.44 - used to normalised the feature counts

10.11. p.11 l.44 - ggpubr - needs to be italicised for consistency

R: We fixed these typos and ambiguities.

Reviewer #2

The study by Agostini et al attempts to stratify the vast amount of the long non-coding (lnc) RNA transcription in the human genome into two main classes - (1) lncRNAs associated with known genes and thus representing products of (by the authors definition) "fuzzy" transcription from those genes and (2) stand alone lncRNA units. To accomplish this task, the authors have analyzed a large amount of publicly available transcriptome and chromatin data.

Conceptual comments:

1. After reading the paper, I am confused about what the authors aim to accomplish and what their main conclusions are. Overall, the logic is very confusing in the text. For example, in terms of the conclusions, in the Introduction, they state that "We conclude that most of the unannotated intergenic RNAs are the consequence of non-productive transcription associated with known genes, which are rapidly removed through cellular quality control mechanisms." However, in Discussion's section "Functional consequences of non-canonical transcription on canonical genes" they describe in details potential functional consequences of the gene-associated transcription. Therefore, even the main conclusion from the paper is not exactly clear.

R: We apologise for the confusion. In our study, we find that an unexpectedly major source of intergenic RNA is derived from transcription of coding genes that continues beyond the annotated genes rather than from lncRNA genes, which we find absolutely striking. Accordingly, our analyses indicate this transcriptional process per se likely has a major impact on chromatin regulation, even though it does not produce functional RNAs from intergenic space, and this also represents the goal of our work. We changed the text accordingly to harmonise the introduction and conclusion of the manuscript.

2. However, even more importantly, it is not quite clear what the authors are trying to achieve here. If the main goal is to characterize such features as association of the lncRNA transcription with protein-coding genes, chromatin- and nuclear-localization and their stability as well as some others, then all this has been already shown in multiple previous studies - see for example PMIDs 28017589, 27870833, 23006825, 26190259, 22955620 just to list a few. In this regard, the authors do not seem to provide novel insights.

R: We understand that, often, studies of intergenic transcription are heavily biased towards lncRNA biology, to such an extent that it comes natural to approach the topic from the lens of lncRNA field. However, we find that annotated lncRNAs represent only a minor proportion of intergenic RNA biology, and we have now annotated intergenic RNA from the perspective of continuous transcription units. As mentioned in the previous point, we find our work of great importance, because it indicates that most intergenic RNA is not functional as an independent transcript, but rather the outcome of transcription of canonical loci . Some systematic studies of lncRNAs have speculated the same (i.e. that lncRNAs themselves are often not functional), but this is a very contentious question that is hard to disentangle. Our finding on the pervasiveness of ongoing transcription from coding genes, which has so far been overlooked, gives a more straightforward evidence in support of the likely importance of transcriptiondependent chromatin regulation.

3. On the other hand, if the authors are trying to use the association with genes as a mean to annotate the lncRNAs in terms of their biological relevance, then this approach is inherently flawed. First and foremost, association with known genes per se can not be used as the evidence of absence of biological function. For example, numerous studies have shown that intronic transcripts - the quintessential geneassociated transcripts - represent a rich source of potentially-functional RNA species as summarized in two recent reviews PMIDs 30391089 and 31668856.

R: Again, we are putting forward the concept that most intergenic RNA is not functional but, in this case, the process of intergenic transcription itself represents the functional outcome.

4. If the authors are trying to provide some sort of a classification scheme to sort lncRNAs into potentially functional or not based on their association with genes, then the results presented are too preliminary and insufficient to address this. At the very least, the authors should provide functional validations of their findings to show that ranscripts that are classified as non-functional in their studies indeed do not have functions and vice versa.

R: As described earlier, the aim of our study is to annotate sources of intergenic RNA, and we find most such RNA is derived from protein coding genes, rather than lncRNAs (i.e. the focus of our study is not on lncRNAs). Keeping this in mind, we have validated the transcriptional state of our assigned genomic loci by data derived from multiple orthogonal techniques (e.g. NETseq, CAGE, ChIPseq, etc.). In addition, we showed that distinct components of the cellular quality control mechanisms coordinate degradation of different types of loci, some following the exosome-mediated pathways similarly as annotated lncRNAs, and others the Xrn-mediated degradation that is believed to be co-transcriptional. While this indicates limited functionality of the annotated loci, it is very difficult (close to impossible) to ever fully rule out any functionality in biology given that our knowledge is inevitably limited. As the reviewer is surely aware, the functionality remains unclear even for most annotated lncRNAs, so it would be beyond the scope of our study to classify functions of the newly annotated loci.

5. The validation work included in the manuscript is not clear. For example, why did the authors choose a separate cell line for the validation considering that lncRNA transcripts are well-known to be cell-type specific? What was the rationale for using HeLa for this purpose? Exactly what the authors were validating and how? All this is not clear.

R: Again, we should first highlight that we are not focusing on lncRNA in the manuscript. HeLa cells are not a “separate cell line used for validation”, but instead they are the cell line with the largest number of independent datasets available, as it is stated at the beginning of the “Gene-associated transcription breaks gene boundaries” section. We therefore selected HeLa for the initial annotation and for cross-validation and analysis with all types of orthogonal data, and we only used HCT116 for the consequences of XRN2-depletion because this particular HeLa dataset found in public repositories was of poor quality. For validation, we assessed transcriptional status of the TUs (and associated genes) with independent techniques and datasets (i.e. not used for annotating the features), and their stability upon knockdown of several nucleases. We have now emphasised in the revised version that our validation is focused on HeLa data, which of course expresses only a subset of TUs. Nevertheless, we did take the step to account for cell-type specific expression by using data from cells derived from different human tissues (i.e. HeLa, HEK293, HepG2, K562, HCT116 and B cells) to further generalise the annotation of TUs, in order to provide a more comprehensive annotation database of use to those interested in cell types from various tissues.

Technical comments:

1. The authors provide multiple comparisons between chromatin, CAGE and RNAseq data. However, its not immediately clear if these comparisons are valid. Some of these datasets are based on a very broad numbers of cell types. However, it is a well-known fact that expression of lncRNAs is much more cell-type specific compared to the protein-coding mRNAs. Are the authors limiting their comparison to the same cell type or are they comparing bulk data? If the latter, the lncRNA-associated chromatin or CAGE signal can be easily lost.

R: This is clearly stated in the Methods “Selection of HeLa TUs and metadata profiles” section: although the annotation was performed using multiple cell lines, the validation of transcriptional and genomic features has been carried out on a HeLa-specific datasets (i.e. TUs that are expressed above 1 TPM in HeLa cells). With the exception of the robust phase 1 and 2 CAGE peaks, which were defined by the FANTOM5 consortium using bulk data, all comparisons were performed using datasets derived specifically from HeLa cells.

2. The pipeline presented in Figure 1 describes only the basic analysis of the RNAseq data and does not show how the lncRNA transcripts were classified into the different classes. The authors cite custom pipelines in the text without providing any details and any rationale for choosing specific parameters for this classification.

R: We have now provided the github repository to the code (https://github.com/luslab/IntergenicTranscriptionManuscript). The parameters used are either specified in figure 1 or in the Methods section, and the procedure to classify the intergenic regions into different annotation groups is clearly described in the main text (page 3) and Methods section (page 10).

Conclusion:

Overall, the manuscript offers no sufficiently-novel insights and contains too many flaws making it unsuitable for publication in the present form.

**2^nd^ round**

**Reviewer 1**

I am satisfied with the author's answers to my previous concerns, and I think this manuscript follows a rigurous methodology to describe and characterize intergenic transcripts. In my opinion, it complements the results from the van Bakel study.

Minor comments:

The authors should include citations to both CHESS and gffcompare as they both have associated publications that appeared within the last couple of years.
